# Ceria/silicon carbide core–shell materials prepared by miniemulsion technique

**DOI:** 10.3762/bjnano.2.67

**Published:** 2011-09-27

**Authors:** Lars Borchardt, Martin Oschatz, Robert Frind, Emanuel Kockrick, Martin R Lohe, Christoph P Hauser, Clemens K Weiss, Katharina Landfester, Bernd Büchner, Stefan Kaskel

**Affiliations:** 1 Department of Inorganic Chemistry, Dresden University of Technology, Bergstrasse 66, D-01062 Dresden, Germany; 2 Leibniz Institute for Solid State and Materials Research Dresden (IFW Dresden), Institute for Solid State Research, Helmholtzstrasse 20, 01069 Dresden, Germany; 3 Max Planck Institut für Polymerforschung, Ackermannweg 10, D-55128 Mainz, Germany

**Keywords:** ceria, cerium dioxide, core shell, miniemulsion, oxycarbide, silicon carbide, TPO catalytic

## Abstract

For the first time we present the synthesis of CeO_2_/Si(O)C core–shell particles prepared by the miniemulsion technique. The Si(O)C core was obtained by means of a polycarbosilane precursor (SMP10), which was subsequently functionalized with ceria and pyrolyzed to the ceramic. The size of these particles could easily be adjusted by varying the surfactants and the surfactant concentration, or by the addition of comonomers. Hence particle sizes ranged from 100 to 1000 nm, tunable by the preparation conditions. All materials were characterized by photon cross correlation spectroscopy, scanning electron microscopy and elemental mapping investigations. Furthermore, first catalytic tests were carried out by temperature programmed oxidation (TPO) of methane, and the activity of this material in lowering the onset temperature of methane combustion by 262 K was documented.

## Introduction

In recent years miniemulsions have been studied intensively [1–3]. Polymeric nanoparticles [1–2] from homo- or copolymers [3] as well as hybrid materials [3–4] such as magnetic [5–8] or silica/polymer nanoparticles [9–10] have been synthesized by this approach. The size of the generated particles can easily be controlled [11–12] through the amount of surfactant added to the system, allowing particle sizes usually in the range of 50–500 nm and with a narrow size distribution. Hydrophobic polymeric particles are usually prepared from a direct (oil-in-water) miniemulsion, with the monomer as the dispersed oil phase. The nanodroplets are generated by shearing this system with ultrasound. A highly hydrophobic osmotic pressure agent (costabilizer) is added to the oil phase, effectively suppressing diffusional degradation (Ostwald ripening) of the droplets. Thus, the droplet sizes and the composition of the droplet components remain unchanged. This, in consequence, enables the preparation of copolymer particles of defined composition and the encapsulation of further, monomer soluble materials [4]. For the preparation of inorganic, ceramic materials usually the inverse miniemulsion technique has to be applied. Here, water soluble precursor compounds (e.g., Ti- or Si-glycolates, Zr or Ce-salts) for sol–gel synthesis and, if desired, templating surfactants, such as CTAB, are dissolved in water, acting as the dispersed phase. After miniemulsification and sol–gel reaction, porous oxide nanoparticles are obtained [13–17]. However, miniemulsions can also be useful for the synthesis of nonoxide ceramics, such as carbides or nitrides, which can serve as catalysts or catalyst support for highly exothermic or high temperature reactions. Important requirements concerning these materials are chemical inertness and temperature stability.

A material with high temperature stability, as well as excellent heat conductivity, hardness and mechanical stability is SiC [18]. Next to bulk SiC, also composites [19], porous [20–25], and nanosized [26] silicon carbide are becoming increasingly interesting. There are several reports in literature showing that these materials are able to compete with supports such as alumina, silica or activated carbons, particularly in exothermic reactions [27–30].

In particular, the use of polymeric precursors for the synthesis of SiC ceramics (polymer derived ceramics) [31–32] has been found to be an easy approach. Herein, we report the synthesis of nanosized silicon(oxy)carbide spheres by the miniemulsion technique with the aid of a polycarbosilane precursor. The first studies using this approach were reported by Kroke et al. [33]. Here we present a new method to achieve catalytic functionalization and control of the particle size for these spheres either by using different surfactants, surfactant concentrations or by copolymerization with comonomers such as styrene (Sty), methyl methacrylate (MMA) or acrylic acid. Furthermore the prevalent problem of sphere sintering during pyrolysis has been overcome by means of a coating procedure. In this contribution, we describe the functionalization of SiC spheres with ceria shells. Ceria is known as an oxidation catalyst for soot combustion reaction [34–35]. Thus, we report for the first time a CeO_2_/SiC core–shell system with tunable particle sizes through a miniemulsion technique, and demonstrate its use as a catalyst for the oxidation of methane.

## Results and Discussion

**Figure 1:**
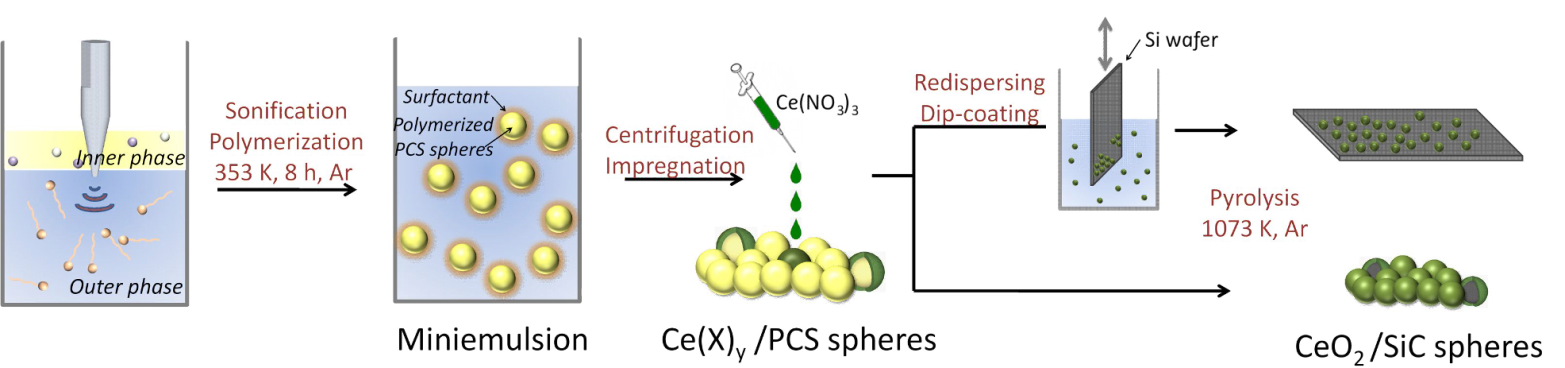
Scheme of the synthesis of CeO_2_/Si(O)C core–shell nanoparticles via miniemulsion technique.

Polycarbosilane (PCS) nanospheres were synthesized from a miniemulsion of SMP-10 (allylic functionalized polycarbosilane) in water (Figure 1). In order to demonstrate efficient tailoring of the sphere size, we used several surfactants in varying concentration. The addition of comonomers was investigated with regard to their effects on particle sizes.

**Figure 2:**
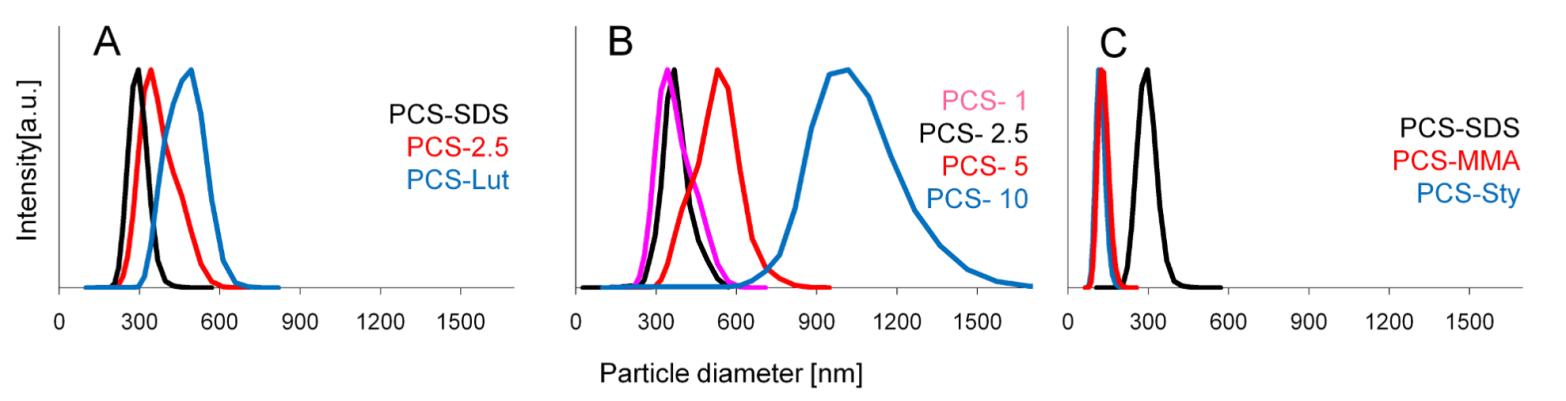
Photon cross-correlation spectroscopy (PCCS) measurement of PCS/water miniemulsions with A) different surfactants, B) different concentration of CTAB surfactant and C) different comonomers.

The results of photon cross-correlation spectroscopy (PCCS) reveal that PCS-spheres synthesized with 2.5 wt % (with respect to the inner phase) of the cationic surfactant cetyl trimethylammonium bromide (CTAB) or the anionic surfactant sodium dodecyl sulfate (SDS) have diameters of approximately 300 nm, whereas the use of nonionic Lutensol AT50 results in larger spheres of 500 nm (Figure 2A).

**Figure 3:**
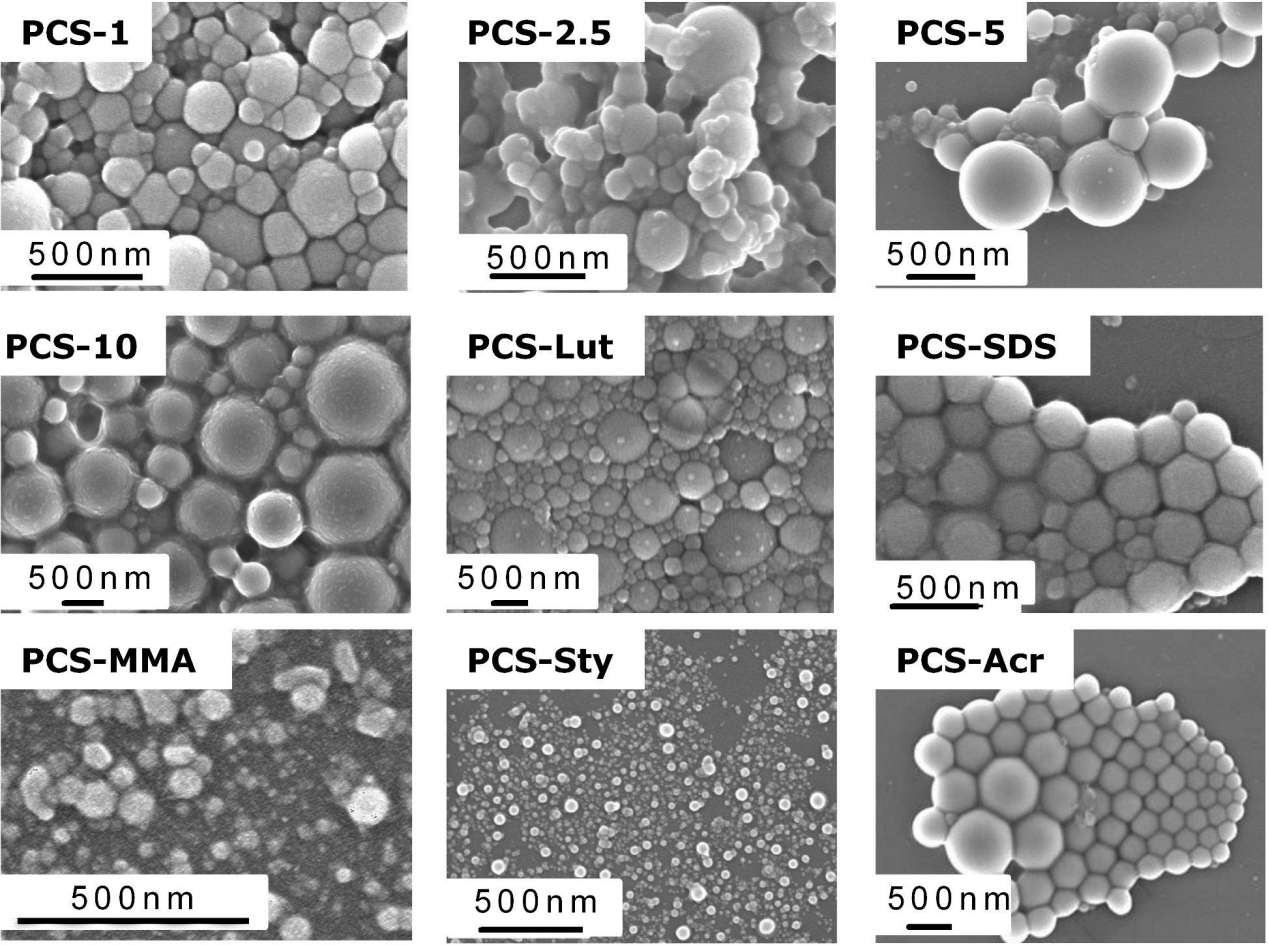
Scanning electron micrographs of PCS spheres synthesized with different amounts of CTAB (PCS-1 - PCS-10), different surfactants (PCS-Lut, PCS-SDS) and comonomers (PCS-MMA, PCS-Sty).

This is not surprising, as nonionic surfactants are less effective in stabilizing colloids. Thus a larger amount of nonionic surfactant is required to achieve the same particle size as with an ionic surfactant. The variation of SDS concentration in the range of 1–10 wt % does not influence the particle size, but in the case of CTAB an increasing amount of surfactant leads to increasing sphere sizes. This is contrary to our expectations, but FESEM (Field Emission Scanning Electron Microscopy) investigations verified that at high CTAB concentrations large particle aggregates are formed. Elucidation of the particular mechanism behind this effect is part of the current studies, but we assume that SMP-10 partially hydrolyzes during the synthesis, creating negative charges on the particle surface which may form ion pairs with the positively charged cetyl trimethylammonium cation, thus compensating the surface charges. Nevertheless it must be stressed again that it is possible to control particle sizes in a range of 300–1000 nm by varying the CTAB concentration. Furthermore we showed that for the synthesis of smaller particles the addition of comonomers is useful. The sizes of PCS spheres prepared with 50 wt % of styrene or MMA were reduced to 100 nm (surfactant 2.5 wt % SDS) (Figure 2C). Particles sizes as well as their elemental distribution were very uniform, indicating that copolymerization had occurred. The addition of acrylic acid did not influence the size of the resulting PCS spheres. Scanning electron micrographs verified all these trends but also showed that the PCS spheres synthesized with SDS (PCS-SDS, PCS-Sty, PCS-MMA) exhibited a narrower distribution of particle sizes than those synthesized with CTAB or Lutensol AT50 (Figure 3). The green PCS spheres exhibited a low specific surface area of ~9 m^2.^g^−1^.

### Functionalization and pyrolysis

The dispersion of PCS spheres can be destabilized either by adding acetone, by the evaporation of water at 353 K overnight, or by centrifugation. Subsequently, the resulting PCS spheres are either pyrolyzed instantaneously or functionalized before pyrolysis. The latter results in a core–shell-structured hybrid material. A promising method for the synthesis of core–shell hybrid materials in general was described by Landfester et al. [36]*.* They created surface functionalized polymer spheres coated with hydroxyapatite. Accordingly, we used the surface functionalized PCS/acrylic acid spheres for the growth of a CeO_2_ shell. Additionally, dip coating of the unfunctionalized PCS spheres in an ethanolic Ce(NO_3_)_3_ solution was investigated. Functionalized as well as unfunctionalized PCS spheres were pyrolyzed at 1073–1573 K. Preliminary investigations showed that a simple bulk pyrolysis of PCS spheres, especially at high temperatures, either leads to particle aggregation or to large amounts of sintered spheres being obtained, which lose their spherical shape. Therefore pyrolysis was additionally performed on a silicon wafer in order to obtain single particles. All samples were X-ray amorphous, which is in agreement with the fact that crystalline SiC is usually generated from SMP-10 precursors at temperatures above 1573 K [22].

**Figure 4:**
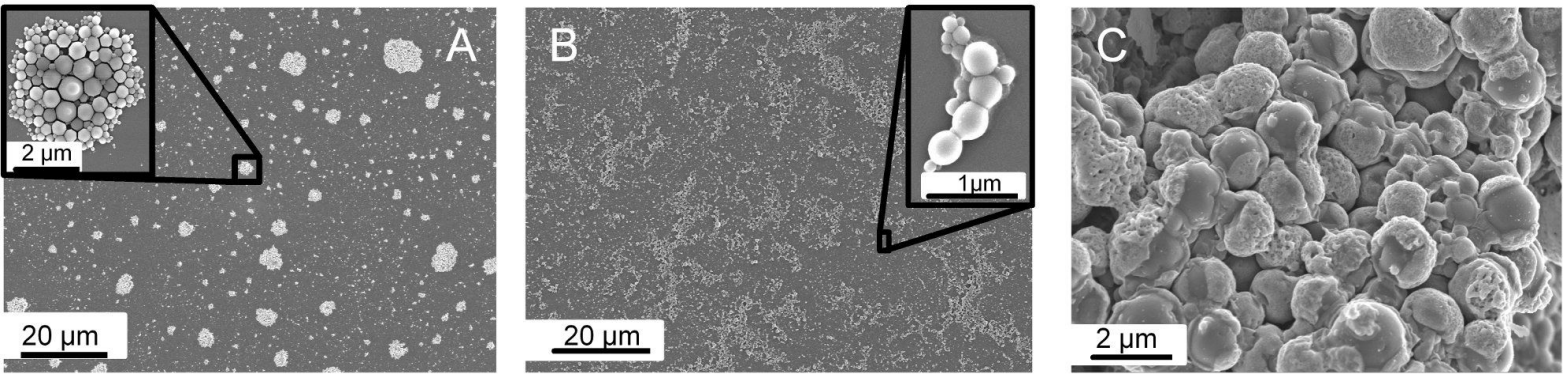
SEM micrographs of (A) unfunctionalized SiC-SDS spheres, (B) SiC-Acr/CeO_2_ spheres prepared by molecular bonding approach and (C) SiC/CeO_2_ spheres prepared by impregnation.

Figure 4A shows the individual particles and illustrates that the shape of the PCS spheres was conserved during pyrolysis. Figure 4B shows SiC-Acr spheres synthesized from PCS/acrylic acid. The carboxylate groups were used for molecular binding of ceria [36]. Although a CeO_2_ shell cannot be seen on SEM pictures, the EDX-analysis of the discrete spheres confirms the presence of cerium (1.5 wt % Ce). Furthermore the catalytic tests, shown in the next chapter, prove the presence of ceria. The core–shell structure could be seen more clearly when CeO_2_/Si(O)C particles that were synthesized by an impregnation approach were considered. From the scanning electron micrographs an average shell thickness of approximately 60 nm was obtained. Figure 4C illustrates the formation of these ceria shells on silicon carbide spheres. The cerium loading of these materials was increased up to 4 wt % Ce.

**Figure 5:**
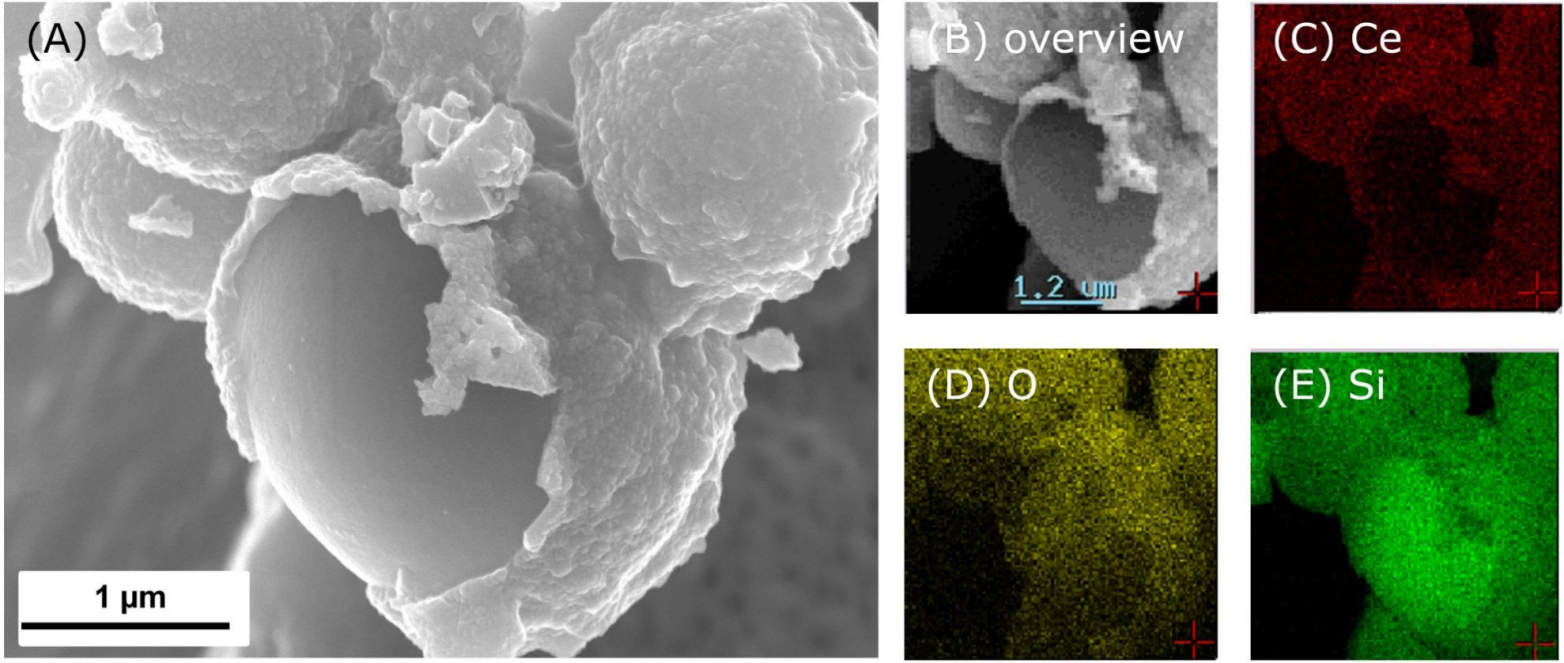
Elemental mapping investigations on CeO_2_/Si(O)C core–shell nanoparticles prepared by impregnation.

Element mapping with EDX was used in order to verify the core–shell structure. To achieve this, a sphere with a partially fractured shell (Figure 5A) was analyzed with regard to the distribution of cerium, oxygen and silicon. Figure 5C proves that cerium is only present in the shell of this hybrid material. The shell also contains a higher amount of oxygen than the core. The presence of oxygen at the inner sphere part can be explained by the formation of Si(O)C, which is well known for polymer derived silicon carbides [37]. It should be noted that oxygen impurities can also be introduced into the SiC core through the partial hydrolysis of the polycarbosilane precursor during the miniemulsion step, which is carried out in aqueous solution. The distribution of silicon is shown on Figure 5E. The overall composition of these core–shell particles is 4 wt % Ce, 19 wt % O, 44 wt % Si and 33 wt % C. As this data only hints at the presence of Ceria and the amounts are too small for detection in X-ray diffraction experiments, TEM investigations were carried out on different samples.

**Figure 6:**
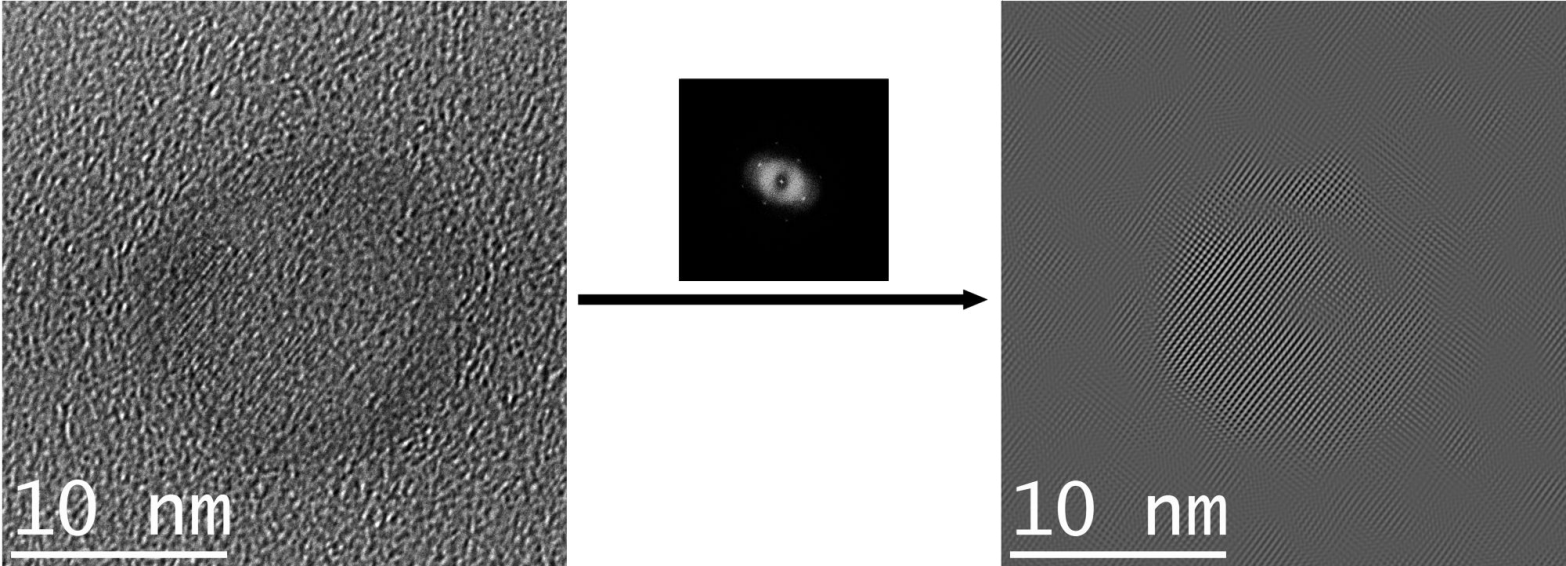
TEM image of a cerium oxide particle (left) with the corresponding diffractogram (middle) and a filtered image for better visibility of the lattice fringes (right).

The cerium oxide particle phases were determined by comparing the lattice spacings measured from the TEM images with literature data. It can be shown that CeO_2_ is present in the samples, but also phases with less oxygen, such as Ce_2_O_3_, can be found. In Figure 6 an image of a CeO_2_ particle, the corresponding FFT and a filtered image are shown. The lattice spacing was determined in fourier space for the strongest peak and found to be 2.59 Å which is in good agreement with the data given for CeO_2_ (2.60 Å, ICDD, No. 44-1001).

### Catalytic methane combustion

**Figure 7:**
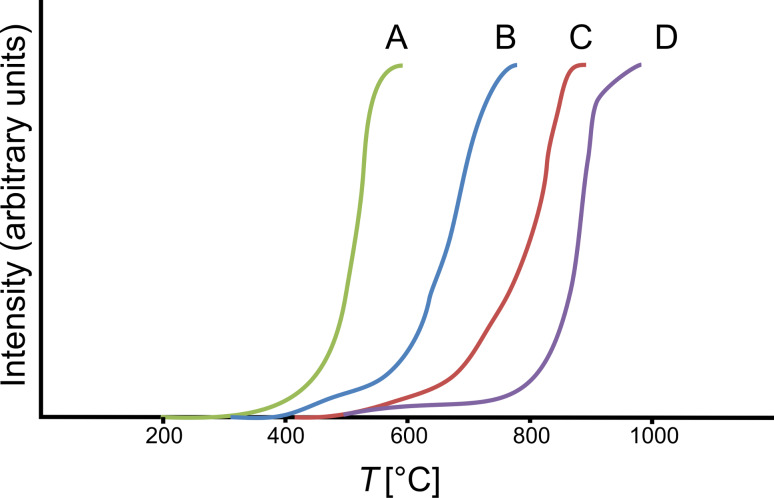
TPO measurements of CeO_2_ nanoparticles (A), SiC-Acr/CeO_2_ (B), SiC/CeO_2_ (C) and the unloaded SiC shell, not containing Cerium (D).

The catalytic activity was determined by temperature programmed oxidation (TPO) of methane. TPO was performed on SiC/CeO_2_ and SiC-Acr/CeO_2_. For comparison of the results, CeO_2_ nanoparticles that were precipitated from aqueous solution and the unloaded Si(O)C shell particles were chosen. The results of the TPO measurements are presented in Figure 7. The onset temperatures for pure ceria nanoparticles and the unloaded particles, representing the uncatalyzed reaction, are 758 K and 1130 K, respectively [38]. The investigated ceria modified Si(O)C spheres show catalytic activity for the combustion of methane. The onset temperatures for SiC/CeO_2_ and SiC-Acr/CeO_2_ are 1018 K and 868 K, respectively. In comparison to the ceria nanoparticles the activity is lower due to the smaller amount of active material in the sample. Although SiC-Acr/CeO_2_ has a smaller amount of active material (1.5 wt % Ce) than SiC/CeO_2_ (4 wt %), it shows a higher activity, which can be explained by the more efficient immobilization of the cerium nitrate on the acrylic acid modified surface of the PCS spheres during functionalization. The specific surface area of SiC-Acr/CeO_2_ (15 m^2.^g^−1^) is higher than that of SiC/CeO_2_ (<0.01 m^2.^g^−1^), thus this also has to be considered as a contribution to the difference in catalytic activity. The enlarged specific surface area for SiC-Acr/CeO_2_ is attributed to additional amorphous carbon in the spheres resulting from the combustion of acrylic acid during pyrolysis. However, the results are promising; especially considering that only 1.5 wt % of Ce was needed to decrease the onset temperature for methane combustion by 262 K.

## Conclusion

We presented the synthesis of silicon(oxy)carbide spheres by a miniemulsion process. The size of these spheres can be adjusted through the use of different surfactants or surfactant concentrations. For a given surfactant concentration, nonionic surfactants, such as Lutensol AT50, cause larger particles to be formed than do ionic surfactants, such as SDS or CTAB. The increase of the surfactant concentration leads to larger spheres for particles synthesized with CTAB, whereas no influence was detected for spheres synthesized with SDS. The addition of comonomers such as styrene or MMA also lowers the particle size. Green PCS bodies were functionalized in two different ways with a ceria shell and were converted to silicon(oxy)carbide by pyrolysis under an inert atmosphere. The resulting hybrid materials were studied with scanning electron microscopy and elemental mapping, which verified the core–shell design of this new material. Finally, it was shown that these materials are suitable as catalysts for the oxidation of methane.

## Experimental

### Synthesis of PCS spheres

SMP10 (Starfire Systems), comonomer, 30 mg of hexadecane (Fluka, 98%) and 30 mg of AIBN (Fluka, 99%) were mixed and added to solutions of different amounts of surfactant in 30 g water (Table 1). After stirring the mixture for 1 h, miniemulsification was achieved by ultrasonicating the mixture for 120 s with a Branson sonifier W450 Digital at 90% amplitude and 100% cycle. During sonication the mixture was cooled in an ice-bath. The miniemulsion was polymerized by heating to 353 K for 8 h in an argon atmosphere (Figure 1).

**Table 1:** List of polycarbosilane nanospheres prepared by the miniemulsion technique.

**Sample**	SMP-10 [g]	Comonomer	[g]	Surfactant	[g]

**PCS-1**	1.65	-	-	CTAB^a^	0.0165
**PCS-2.5**	1.65	-	-	CTAB^a^	0.0413
**PCS-5**	1.65	-	-	CTAB^a^	0.0826
**PCS-10**	1.65	-	-	CTAB^a^	0.165
**PCS-Lut**	1.65	-	-	AT50^b^	0.0413
**PCS-SDS**	1.65	-	-	SDS^c^	0.0413
**PCS-Sty**	0.825	Styrene^d^	0.825	SDS^c^	0.0413
**PCS-MMA**	0.825	MMA^e^	0.825	SDS^c^	0.0413
**PCS-Acr**	0.825	Acrylic acid^f^	0.825	SDS^c^	0.0413

^a^cetyl trimethylammonium bromide (Acros, 99%), ^b^Lutensol AT50 (BASF), ^c^sodium dodecyl sulfate (Fluka, 99%), ^d^styrene (Acros, 99%), ^e^methyl methacrylate (Merck, 99%), ^f^acrylic acid (ABCR, 99%).

### Functionalization

The miniemulsion was placed in a cabinet dryer for the removal of water. 60 mg of the resulting polycarbosilane (PCS) powder was added to a 0.75 M solution of 1 g Ce(NO_3_)_3_·6 H_2_O (Aldrich, 99%) in 3 mL ethanol, treated in an ultrasonic bath and finally separated by centrifugation.

In case of the surface functionalized PCS-Acr spheres (comonomer = acrylic acid), 3.5 mL of this PCS-Acr miniemulsion was added to an 0.1 M aqueous solution of 440 mg Ce(NO_3_)_3_·6H_2_O (Aldrich, 99%) and stirred overnight at RT. The PCS-nanospheres were destabilized by adding acetone, centrifuged and washed with water.

### Coating and pyrolysis

The functionalized PCS nanospheres were either pyrolyzed as synthesized or coated on a silicon wafer at 1073 K under an argon atmosphere (RT–573 K at 150 K·h^−1^, then 5 h at 573 K, followed by heating to 973 K at 30 K·h^−1^. After reaching 973 K, the sample was heated to 1073 K at 120 K·h^−1^ and maintained for 2 h). In case of coating, the nanospheres were redispersed in EtOH and coated (1.1 mm·s^−1^) onto a silicon wafer by means of a dip coater. Afterwards the wafer with the particles was pyrolyzed at 1073 K under an argon atmosphere.

### Characterization

FESEM (Field Emission Scanning Electron Microscopy) and elemental mapping investigations on polymers and ceramics were carried out with a Stereoscan 260 SEM with EDX analysis system using SE (Secondary Electrons) and BSE (Backscattered Electrons) detectors, respectively. Elemental analyses using EDX were obtained as a mean value of five measurements at a magnification of 3000. TEM investigations were carried out by crushing the synthesized powders in a ball mill, followed by ultrasonically assisted suspension in ethanol or isopropanol. The resulting suspension was dropped onto a copper grid coated with holey carbon and dried using an infrared lamp. The TEM investigations were carried out on a Cs-corrected JEOL JEM-2010F. Particle sizes were determined with photon cross-correlation spectroscopy (PCCS) using a Nanophox particle sizer (Sympatec GmbH). The dispersions were diluted with demineralized water for the measurement. Catalytic investigations were carried out as described in previous studies [38].
